# Damage to Fronto-Parietal Networks Impairs Motor Imagery Ability after Stroke: A Voxel-Based Lesion Symptom Mapping Study

**DOI:** 10.3389/fnbeh.2016.00005

**Published:** 2016-02-01

**Authors:** Kristine M. Oostra, Anke Van Bladel, Ann C. L. Vanhoonacker, Guy Vingerhoets

**Affiliations:** ^1^Department of Physical and Rehabilitation Medicine, Ghent University HospitalGhent, Belgium; ^2^Department of Rehabilitation Sciences and Physiotherapy, Ghent UniversityGhent, Belgium; ^3^Department of Experimental Psychology, Ghent UniversityGhent, Belgium

**Keywords:** motor imagery, stroke, lesion symptom mapping, basal ganglia, white matter

## Abstract

**Background:** Mental practice with motor imagery has been shown to promote motor skill acquisition in healthy subjects and patients. Although lesions of the common motor imagery and motor execution neural network are expected to impair motor imagery ability, functional equivalence appears to be at least partially preserved in stroke patients.

**Aim:** To identify brain regions that are mandatory for preserved motor imagery ability after stroke.

**Method:** *Thirty-seven* patients with hemiplegia after a first time stroke participated. Motor imagery ability was measured using a Motor Imagery questionnaire and temporal congruence test. A voxelwise lesion symptom mapping approach was used to identify neural correlates of motor imagery in this cohort within the first year post-stroke.

**Results:** Poor motor imagery vividness was associated with lesions in the left putamen, left ventral premotor cortex and long association fibers linking parieto-occipital regions with the dorsolateral premotor and prefrontal areas. Poor temporal congruence was otherwise linked to lesions in the more rostrally located white matter of the superior corona radiata.

**Conclusion:** This voxel-based lesion symptom mapping study confirms the association between white matter tract lesions and impaired motor imagery ability, thus emphasizing the importance of an intact fronto-parietal network for motor imagery. Our results further highlight the crucial role of the basal ganglia and premotor cortex when performing motor imagery tasks.

## Introduction

Motor imagery can be defined as a dynamic state during which a person mentally simulates a given action without actually performing it (Decety and Jeannerod, 1996). Mental practice is the cognitive process through which a person repeatedly mentally rehearses a specific task without performing any actual body movement. Mental practice through motor imagery has been shown to promote motor skill acquisition in healthy subjects and in patients (Grouios, 1992; Heremans et al., 2012; Burianová et al., 2013; Kraeutner et al., 2015b). A combination of mental practice and physical practice has been recommended to improve upper and lower limb function in stroke patients and to promote relearning of daily tasks in neurologic rehabilitation (Liu, 2004; Page et al., 2007; Malouin and Richards, 2010; Schuster et al., 2011; Cho et al., 2012). Especially patients in an early stage after stroke may benefit from motor imagery training. Mental practice can re-activate sensorimotor networks and induce neuroplasticity, thus preventing maladaptive non-use reorganization (Butler, 2006; Lotze and Cohen, 2006; Page et al., 2009).

Despite general optimism, recent negative trials have shown that not all patients may benefit from mental practice (Ietswaart et al., 2011; Timmermans et al., 2013). Ietswaart et al. (2011) investigated the effect of 4 weeks of mental practice without any specific related physical practice in a large cohort of stroke patients. Their results showed that mental practice alone did not enhance motor recovery in stroke patients early post-stroke. As a result, more information about the relationship between motor imagery ability, brain damage, and motor training outcome remains warranted. Neuroimaging studies have revealed that imagination of an action and actual motor execution share many common motor and motor-related regions (Gerardin et al., 2000; Sharma et al., 2009; Szameitat et al., 2012; Kraeutner et al., 2014). Motor imagery and physical practice networks are not completely overlapping but appear to be functionally equivalent (Lotze and Halsband, 2006; Di Rienzo et al., 2014).

The parts of the neural system that are most frequently reported to be involved in motor imagery are the supplementary motor area, the premotor area, posterior parietal regions, the basal ganglia and the cerebellum (Munzert et al., 2009; Liepert et al., 2012; Hétu et al., 2013).

Several investigators have explored motor imagery ability in stroke populations. Due to the loss of integrity of the motor planning network (including premotor, posterior parietal, and prefrontal regions), motor imagery ability is expected to be impaired accordingly in these patients. Damage to the parietal cortex has been shown to impair the generation of movement images (Sirigu et al., 1996; McInnes et al., 2015).

However, research provides evidence that motor imagery ability is at least partially preserved in most stroke patients with motor imagery profiles paralleling actual motor impairments (Lotze and Halsband, 2006). In a study by Liepert et al. (2012) it was shown that patients with somatosensory deficits were more impaired in their ability to perform a mental chronometry task than stroke patients with pure motor deficits. The impairment of mental chronometry seemed to be the result of a reduction in somatosensory input from the affected upper limb. Malouin et al. (2008) found that the level of motor imagery vividness following stroke was similar to that of healthy subjects with good and bad imagers in both groups, although they reported a better motor imagery vividness for the unaffected side. Using the Movement Imagery Questionnaire-Revised second version (MIQ-RS), we have shown in a previous study that both the visual and kinesthetic imagery modalities differed significantly from normal data in our stroke study cohort. However, motor imagery vividness responded well to motor imagery training with a significant increase in kinesthetic subscale scores (Confalonieri et al., 2012; Oostra et al., 2015). confirmed the shared neural circuitry between motor imagery and motor execution, involving a widely distributed frontoparietal network and subcortical structures, in chronic stroke patients. Moreover, low kinesthetic imagery ability was correlated with more activation of the contralesional primary motor cortex and ipsilesional primary somatosensory cortex in their study cohort. Currently, lesions in the parietal cortex, left prefrontal area and basal ganglia have been reported to result in a loss of motor imagery ability in stroke patients (Sirigu et al., 1995; Li, 2000). In an extensive review (Di Rienzo et al., 2014) recently concluded that cerebral activity during motor imagery is highly correlated to structural and functional neuroplasticity. However, a specific lesion localization that was unequivocally correlated with impaired motor imagery ability could not be identified. Therefore, our goal in the present study was to clarify which brain regions are necessary for intact motor imagery ability after stroke. To evaluate motor imagery ability two behavioral tests were combined. To measure motor imagery vividness—both the clarity/sharpness of images and the intensity of sensations during motor imagery—a motor imagery questionnaire was used. The ability to preserve the temporal characteristics between the physical movement and motor imagery movement was measured using a temporal congruence test (McAvinue, 2008). We examined the relationship between perceived motor imagery vividness of stroke patients, reflected by their score on the MIQ-RS and brain lesion localization on the one hand and temporal congruence between real and imagined movements and brain lesion localization on the other. To test whether impaired motor imagery ability was significantly associated with certain lesion locations in the brain, we conducted a voxel-based lesion-symptom mapping analysis, a technique that statistically assesses the lesion's affect on behavioral scores on a voxel-by-voxel basis (Bates et al., 2003). The technique allows us to conduct a statistical test in each lesioned voxel to determine if a difference exists between the lesioned and non-lesioned group for a certain behavioral measure (Rorden et al., 2007).

## Materials and methods

### Method

#### Participants

Thirty-seven patients with hemiplegia after a first time stroke participated in this study. Twenty-five males and twelve females were included. The average age was 53 years with a range of 17–68 years. The time from stroke varied from 1 to 12 months, with a mean disease duration of 4 months. Most patients (*n* = 34) underwent formal neuropsychological testing. Rather than presenting all these data, we selected a measure of attention, the Test of Attentional Performance, as marker of general information processing speed as this ability is likely to be most reflective of the cognitive demands required by the behavioral tasks in this study. The Test of Attentional Performance comprises simple reaction time paradigms with the patient reacting selectively to non-verbal stimuli with a simple key-press (Zimmermann et al., 2012). The results of this neuropsychological test are presented as Z-scores in Table 1. The average Z-score of 0.86 reflects that most patients performed within one standard deviation (SD) below the normative mean on the attention task. Only five patients performed more than 2 SD below the norm and no one exceeded the 3 SD limit. To measure motor recuperation the Fugl-Meyer Assessment Scale was used. This instrument measures distinct parameters of motor recuperation such as reflexes, voluntary control of isolated movement, co-ordination, speed, and balance. The lower-extremity component of the scale consists of a total score of 34 points, the upper-extremity component comprises a total score of 66 points with all items scored on a 0–2 scale (Fugl-Meyer et al., 1975). The results of the Fugl-Meyer scale are presented in Table 1. For a more detailed account of the individual data summarized in Table 1, the reader is referred to Supplementary Table 1.

**Table 1:** **Patients' characteristics**.

**Characteristics**	
Age (years)	53 (range 17–68 years)
Gender (♀:♂)	12:25
Side hemiplegia	
Right	15
Left	22
Cause hemiplegia	
ischemic	21
hemorrhagic	16
Time since stroke (months)	4 (range 1–12 months)
Fugl-Meyer Assessment Scale Upper Extremity (/66)	30.1 ± 10.3 (mean ± SD)
Fugl-Meyer Assessment Scale Lower Extremity (/34)	19 ± 6.2 (mean ± SD)
Test of Attentional Performance	−0.86±0.9 (Z-score, mean ± SD)

The patients were recruited via the University Hospital and from hospitals in East and West Flanders to the Rehabilitation Centre, Ghent University Hospital. Participants were eligible if they: (1) had experienced a first time stroke less than 1 year before entering the study; (2) were between 16 and 70 years old and (3) did not suffer from psychiatric symptoms or any other neurological disease.

All subjects provided written informed consent according to the Declaration of Helsinki. This study was approved by the Ethical Committee of the Ghent University Hospital (registration n° B67020084961).

## Materials and procedures

### Motor imagery ability

Motor imagery vividness was assessed using a self-report questionnaire, developed and validated by Hall et al. in order to assess visual and kinesthetic modalities of movement imagery (Hall, 1997). A revised version, the MIQ-RS was developed by Gregg et al. (2010) for use in people with limited mobility and validated by Butler et al. (2012) for evaluating motor imagery ability in stroke populations. The MIQ-RS is composed of 2 sub-scales of 7 relatively simple movements (like bending forward or pulling on a door handle). For each item, 4 steps are required. First, the starting position of the movement is described by the examiner and the subject is asked to assume it. Secondly, the movement is described and the subject is asked to perform it. Thirdly, the subject is asked to reassume the starting position and imagine producing the movement (no actual movement is made). Finally, the subject is instructed to rate the ease/difficulty with which he/she imagined the movement on a 7-point scale, where 1 is very difficult and 7 is very easy to picture (the visual sub-scale, MIQ-RS_vis_) and to feel (the kinesthetic sub-scale, MIQ-RS_kin_).

When administering the kinesthetic subscale, subjects were encouraged to imagine the tasks from a first person perspective and feel themselves moving. For the visual subscale, the subjects were instructed to see themselves moving from a third person perspective as if looking from a distance to themselves. Mental chronometry tests measure the temporal coupling between real and imagined movements and evaluate motor imagery accuracy. The temporal congruence test was developed by Malouin and co-workers and measures the temporal correspondence between imagined and actual stepping movements (Malouin, 2008). The patients are seated in a chair and instructed to first imagine and then to physically perform 5 stepping movements, placing their foot on a board in front of them. During the imagery task, the subjects have their eyes closed. The examiner records the duration of real and imagined stepping movements. According to the mental chronometry paradigm, it is expected that movement times in both conditions will be similar with the imagined/actual movement time ratio equalling one.

### Analysis of imaging data

Structural brain images were obtained using MRI scans, which were performed on clinical indication in the sub-acute phase after stroke. Thirty-one patients were scanned in the Ghent University hospital on 1,5 T Siemens Trio scanners (Siemens, Erlangen, Germany). Due to initial hospitalization in another setting, the remaining 6 patients were scanned with 1.5 T MRI scanners in different hospitals. For each subject a whole brain T1-weighted anatomical image that was obtained in the sagittal orientation and a FLAIR image in the transverse plane was available. Clinical T1 images were scanned with a pixel spacing around 0.45 × 0.45 mm and slice thickness between 4 and 6 mm, clinical FLAIR volumes were scanned with a pixel spacing around 0.9 × 0.9 mm and slice thickness between 4 and 6 mm. Brainvoyager software was used for MRI data processing and normalization (Goebel, 2012). All T1 and FLAIR scans were isovoxelized to a 1 × 1 × 1 mm resolution using sinc interpolation. We used sagittal T1 MPRAGE images for AC-PC orientation of the isovoxelized anatomical scan. Following co-registration of the isovoxelized T1 and FLAIR 3D volumes, AC-PC transformations were applied to the FLAIR scan and this transverse scanned volume was warped into standard Talairach space. Lesion demarcation was based on the FLAIR images that were uploaded in MRIcron. The lesioned areas were manually traced by the first author, using MRIcron to draw the regions of interest (Rorden and Brett, 2000). The extension and location of the lesion shapes were controlled by an experienced radiologist, who was blinded to the performance of the subjects on the motor imagery ability tests. We applied Voxel-Lesion Symptom Mapping analyses to the lesion and behavioral data (Rorden et al., 2007). We used the non-parametric Brunner Munzel test with the significance level set to *p* < 0.01. In the current study, an “a priori” minimum lesion density threshold was set at 20%, i.e., analyses were confined to those voxels in which there were at least 7 patients with and 7 patients without a lesion, in order to avoid running analyses in voxels in which very few patients had lesions. To define a “significant” voxel, a statistical threshold cut-off was determined based on permutation testing (*n* = 2000). With permutation testing the patients' behavioral scores are randomly reassigned across the voxels 2000 times. For each permutated dataset, the statistics are re-run and the top 5% of *t*-values calculated. Brain regions corresponding to the significant voxel locations were determined using the Talairach and Montreal Neurological Institute and Hospital (MNI) co-ordinate systems where appropriate. For the identification of white matter structures, a white matter reference atlas was used (Oishi et al., 2011). All reported coordinates are presented in MNI co-ordinates in **Table 3**.

## Results

### Behavioral results

The behavioral results from 37 subjects with sub-acute stroke are summarized in Table 2. The vividness of motor imagery was measured using a validated motor imagery questionnaire, the MIQ-RS. The mean total MIQ-RS score was 60.84 (SD ± 20.13). The mean score for the MIQ visual subscale was 32, with a range of 11–49. The mean score for the MIQ kinesthetic subscale was 29 with a range of 8 to 47. We found a significant correlation between kinesthetic and visual MIQ-RS subscales (*r* = 0.82, *p* < 0.001).

**Table 2:** **Performance of stroke patients on behavioral motor imagery ability tests**.

**Measures**	***N***	**Minimum**	**Maximum**	**Mean**	***SD***
**MIQ-RS**					
Kinesthetic scale	37	8	47	32.1	10.4
Visual scale	37	11	49	28.7	10.7
Total scale	37	22	96	60.8	20.1
**TEMPORAL CONGRUENCE STEPPING TEST**					
Ratio IS/AS	37	0.00263	0.7717	0.2506	0.176

MIQ-RS, Movement Imagery questionnaire-revised second version; IS, imagined stepping; AS, actual stepping

According to the MI Ability Assessment Scale a total MIQ-RS score ≤ 48 indicates MI inability, a score between 49 and 73 indicates MI impairment, whereas a score ≥74 indicates preserved MI ability (McInnes et al., 2015). The temporal congruence test scores revealed a statistically significant correlation between imagery stepping time and actual stepping time (*r* = 0.78, *p* < 0.001). The MIQ-RS_tot_ score was not significantly correlated with the results of the mental chronometry test (*r* = 0.004, *p* = 0.98).

### Neuroimaging results

**Figure 1:**
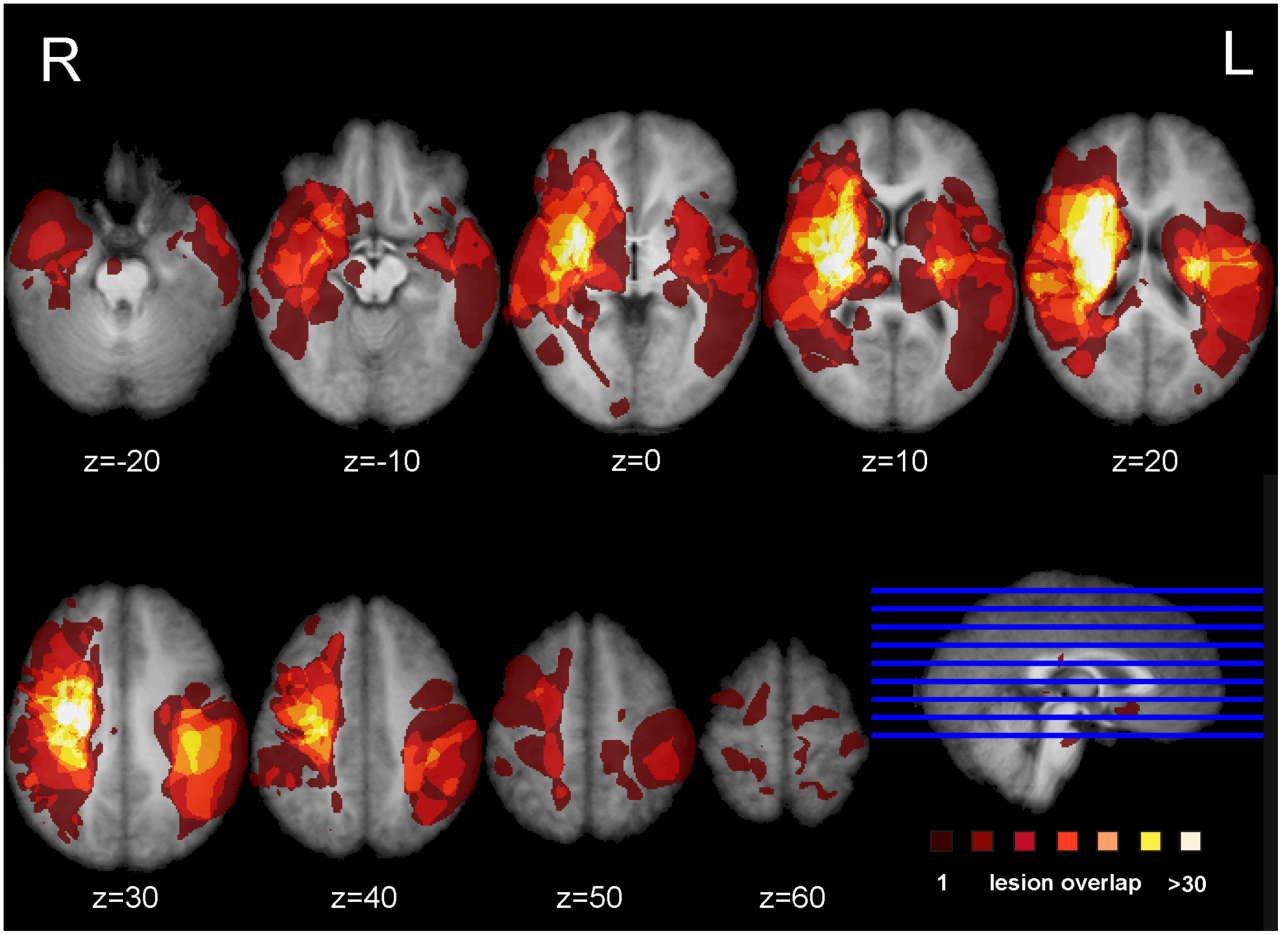
**Lesion overlap map**. Overlap of the binarized lesions of the 37 stroke patients included in the study. Lesions are displayed over horizontal sections parallel to the AC-PC line. Brighter regions indicate a greater degree of overlap of lesions.

Figure 1 shows the lesion overlay map for all 37 stroke patients with brighter regions indicating a greater degree of lesion overlap. Although the distribution of lesions involved both of the hemispheres, lesions and lesion overlap were larger in the right hemisphere where they also encompassed more anterior regions. Significant voxels associated with poor motor imagery vividness were identified in the left hemisphere, with significant foci in the putamen, the left ventral premotor cortex and the underlying white matter, connecting frontal and parietal/occipital regions (superior fronto-occipital fasciculus and claustrum region). The mapping of the temporal congruence score (imagined/actual stepping ratio) on the lesioned brains through VLSM revealed the involvement of an area, localized in the superior part of the corona radiata. The results of the VLSM Brunner-Munzel test are presented in Table 3 below and show significant voxels, to be present in at least 7 patients, marked with MNI coordinates.

**Table 3:** **Results of the VLSM analyses on motor imagery vividness and temporal congruence performance indices**.

**Brain region**	**Hemisphere**	**MNI coordinates**		
		***x***	***y***	***z***
**MOTOR IMAGERY VIVIDNESS**				
Putamen	L	−27	1	−4
Gyrus frontalis inferior pars opercularis	L	−38	−4	3
External capsule/extreme capsule/claustrum^*^	L	−29	−18	13
		−29	−21	14
Superior Fasciculus fronto-occipitalis (sFOF)	L	−20	19	21
		−18	21	18
		−20	11	26
		−22	13	24
		−25	14	21
Anterior Corona Radiata	L	−18	23	18
		−18	27	15
**TEMPORAL CONGRUENCE SCORE**				
Superior Corona Radiata	L	−26	−16	40
		−29	−14	38
		−24	−14	40
		−20	−6	34
		−18	−8	35
		−22	−4	34
		−27	14	21

*Location of significant peak voxels based on MNI coordinates*.

*L, left; x co-ordinate, right/left; y coordinate, anterior/posterior; z coordinate, superior/inferior*.

* *Cannot be resolved with current resolution*.

## Discussion

The present study used a voxel-based lesion-symptom mapping analysis to explore the relationship between motor imagery ability and brain lesion localization after stroke.

Motor Imagery, being an internal process, is inherently difficult to assess in an objective manner. It is difficult to determine to what extent a person is able to generate vivid mental representations of movements and adhere to motor imagery training. Moreover, because motor imagery and motor execution are believed to share the same underlying neural network, any structural damage to the brain could affect both motor performance and motor imagery ability. Although the ability to perform motor imagery seems to be at least partially preserved after stroke, motor imagery vividness and accuracy can be hampered. Sharma et al. (2006) refer to this disturbed motor imagery accuracy as “chaotic” post-stroke motor imagery.

The predictive value of motor imagery ability tests to determine who might be the best potential candidates for mental practice, has not been fully established yet. Moreover, in a previous study we found no significant correlation between initial MIQ-RS scores, changes of the MIQ-RS scores and motor improvement, indicating that poor imagers can equally benefit from motor imagery training (Oostra et al., 2015). However, it seems important to screen for motor imagery ability, to be able to estimate the need to teach motor imagery practice through an individually tailored initiation program, before starting mental practice. We are aware that screening for motor imagery ability on the basis of lesion localization is insufficient, but it may possibly give an indication which stroke survivors are potentially poor imagers.

Our present study results indicate that lesions in the left hemisphere, more specifically the left putamen and left ventral premotor cortex were associated with poor motor imagery vividness in stroke patients as measured with the MIQ-RS questionnaire. We further demonstrated the importance of an intact frontoparietal functional network for motor imagery. More specifically, lesions in the transition area between the anterior corona radiata and the superior fronto-occipital fasciculus and the more ventrally situated fibers near the claustrum were shown to be associated with impaired motor imagery vividness. Poor temporal coupling between real and imagined movements was solely associated with lesions in the white matter tracts, localized in the superior part of the corona radiata of the left hemisphere.

### Basal ganglia

The results of our study further highlight the role of the basal ganglia in motor imagery. We found a correlation between putamen lesions and poor motor imagery ability, a finding which is corroborated by the work of Li (2000). They included patients with lesions of the putamen and compared the results to those obtained in patients with motor cortical lesions. Li found that lesions of the putamen as well as motor cortical lesions impaired movement imagery. Moreover, a recent meta-analysis highlighted that damage to the putamen drives the impairment in MI after basal ganglia damage (McInnes et al., 2015). The role of the putamen in the planning and execution of a self-generated defined action has been demonstrated by several investigators (Monchi et al., 2006; Doyon et al., 2009). Lacourse et al. (2005) provided evidence for a substantial overlap of the functional neuroanatomy maps of movement execution and motor imagery in both early and skilled learning. This investigator showed that congruency between motor imagery and motor execution became increasingly similar in the skilled condition, with putamen activity increasing nearly a hundredfold in the skilled phase of learning. We found significant voxels related to poor motor imagery ability in both the posterior and anterior part of the putamen, with the latter being more robust. Guillot et al. (2008) showed that good and poor imagers differed in putamen function, with poor imagers activating anterior associative regions while good imagers were showing a more posterior activation in the sensorimotor region of the putamen. Finally, the left putamen has been shown to take part in working memory, more specifically the putamen appears to hinder irrelevant information from entering the working memory (Baier et al., 2010). While performing motor imagery, subjects are generating and maintaining an internal model of motor action within the working memory, and the vividness of the imaging experience appears to be associated with the formation and maintenance of the image in working memory. Working memory impairment has clearly been shown to have a negative impact on motor imagery performance (Malouin, 2004).

### The gyrus frontalis pars opercularis (BA 44)

We further defined significant voxels in the left opercular part of the inferior frontal cortex to be associated with poor motor imagery vividness. The crucial role of this area in motor imagery and performance of visually guided movements is in agreement with Binkofski et al. (2000). They demonstrated that a left hemispheric activation of this region was associated with motor imagery from a first person perspective, while the imagery of a moving target was associated with activation of the right ventral opercular cortex. Our aim was to focus on motor imagery from one's own movements and activation of the left ventral premotor cortex (vPMC) was confirmed.

### The superior fronto-occipital fasciculus (sFOF)/claustrum

Our results further revealed a cluster of significant voxels in the transition area between the sFOF and anterior corona radiata that were correlated with poor motor imagery. Prefrontal and parietal regions are shown to form a functional network for motor imagery and disconnections in this network could account for impaired motor imagery ability (McInnes et al., 2015). An activation likelihood estimation meta-analysis by Hétu et al. (2013) demonstrated that a large frontoparietal network is involved when a person imagines himself moving from a first person's perspective. Lorey et al. (2011) further showed that the extent of neural activation of the parieto-premotor areas was closely linked to perceived motor imagery vividness. The sFOF or fronto-occipital fasciculus is one of the long association systems of the dorsal visual stream (Makris et al., 2007). According to Forkel et al. (2014), the sFOF probably represents an occipital extension of the superior longitudinal fasciculus, running in the outermost region of the corona radiata. Our results indicate that lesions in this region of the long associative fibers mediating the integration of visual and sensory information for motor planning and control, are associated with poor motor imagery vividness. Kraeutner et al. (2015a) recently demonstrated the importance of the left inferior parietal cortex (IPL) for MI performance. A preserved function of the IPL was shown to be critical for learning the cognitive aspects of a skill via MI practice.

Vry et al. (2012) describe a similar dorsal network, activated during motor imagery and motor execution in a healthy population. However, they localize the dorsal network more laterally, corresponding to those fibers of the superior longitudinal fasciculus. These authors further describe an imagery-specific left hemispheric network with more ventrally localized fibers converging into the subinsular white matter near the claustrum, assigned to the extreme/external capsule. This finding could be relating to those voxels that we identified in the claustrum region.

Although MI has been shown to activate a widespread and bilateral neural network, we observed in particular a left hemispheric contribution for motor imagery tasks in this study cohort. Left brain dominance for motor planning of complex movements is in agreement with previous findings (Gerardin et al., 2000; Stinear et al., 2007; Bakker et al., 2008). found that activation in the parietal cortex during imagination was predominant in the left hemisphere, while actual movement execution activated both parietal lobes symmetrically. Sabaté et al. (2004) showed an increased performance time in both real and virtual movements in their stroke patients but velocity of imagined movements in both hands only decreased in patients with left-brain lesions.

### Superior corona radiata

In our patient cohort a disturbed temporal coupling between real and imagined movements—as reflected by the results of the temporal coupling test—was solely associated with lesions in the superior part of the left corona radiata. Our results indicate that motor imagery questionnaires and temporal coupling tests address different MI modalities that involve different brain areas. While motor imagery vividness seems to reflect the movement-related anticipatory cognitive processes that precede movement, the temporal congruency between real and imagined stepping movements seems to be more related to pure motor action. Using lesion symptom mapping, Lo et al. (2010) showed that a lesion at the junction of the corona radiata and the corticospinal tract was critical for maintaining complex motor performance in their stroke population. Diffusion Tensor Imaging (DTI) study results identified that this area was connected with the premotor cortex, sensory cortex and primary motor cortex, with fibers converging to form the corticospinal tract, superior to the internal capsule.

### Study limitations

We used the Motor Imagery Questionnaire-Revised second version to classify our patients into high or low imagers. Although MI questionnaires have been shown to be reliable and valid tools to screen for MI vividness and allow us to distinguish between high and low imagers, the scores remain a subjective reflection of the motor imagery capacity of the individual and this subjectivity remains an important disadvantage of this motor imagery measure (Hall, 1997; Guillot and Collet, 2005; Malouin et al., 2008). Nevertheless, a study by Lorey et al. (2011), examining brain activation patterns during the imaging of movements, has shown a close relationship between the motor imagery vividness scores and the level of brain activation.

The use of an implicit motor imagery measure such as the hand laterality test would have been a more objective measure of motor imagery ability. On the other hand, de Vries et al. (2013) demonstrated that implicit and explicit motor imagery were differently affected in stroke patients. The patients in their study cohort scored below controls for both aspects (visual and kinesthetic) of the MIQ-RS while accuracy scores of an implicit motor imagery task did not significantly differ from the control group.

There are further limitations in the interpretations of our study that relate to the involvement and connectivity of white matter tracts. A complementary DTI study, allowing more precise identification and reconstruction of the involved white matter tracts, seems essential to enable us to gain a greater understanding of our findings.

Finally, the small sample is a limitation of this study. Although 37 subjects were included, not all brain regions were sufficiently covered. Only one patient with a cerebellar lesion was included. Given the variability in lesion location coupled with the small sample size, it is possible that the distribution of MIQ scores is not completely reflective of the true distribution that would be observed in a larger sample.

## Conclusion

Our results confirm the importance of an intact functional fronto-parietal network for preservation of motor imagery ability after stroke. Voxel-lesion symptom mapping further identifies the role of the basal ganglia and premotor cortex when performing motor imagery tasks.

## Author contributions

All authors contributed equally to this study. KO: design of the work, gathering data, interpretation of data, drafting the work; AV: gathered data, revised the work critically, final approval; AV: gathered data, revised the work critically, final approval; GV: design of the work, interpretation of data, revised the work critically and approved the final version.

### Conflict of interest statement

The authors declare that the research was conducted in the absence of any commercial or financial relationships that could be construed as a potential conflict of interest.
