# Supplementary material for: Damage to Fronto-Parietal Networks Impairs Motor Imagery Ability after Stroke: A Voxel-Based Lesion Symptom Mapping Study
Source: Front Behav Neurosci. 2016 Feb 1;10:5. doi: 10.3389/fnbeh.2016.00005 (PMC4740776; doi:10.3389/fnbeh.2016.00005)
Supplement: Supplementary file 1 [file Table1.PDF]

Supplementary material

Damage to fronto-parietal networks impairs motor imagery ability after stroke: A voxel-based lesion symptom mapping study.

Kristine M.Oostra* , Anke Van Bladel , Ann Vanhoonacker , Guy Vingerhoets

***Correspondence:** corresponding author: : Kristine.oostra@UZGent.be

Supplementary Table 1. Patients' characteristics.

Patient number	Age (years)	Gender	Time since stroke (months)	Cause hemiplegia	FM-UE Score /66	FM-LE Score /34	TAP visual reaction time Z-score	Aphasia	Brain localization
1	41	F	11	hem	4	8	-2.33	None	R thalamus
2	52	M	3	ischemic	11	18	-0.31	None	R capsula interna
3	51	F	5	ischemic	36	28	-0.31	None	R frontotemp
4	53	M	3	ischemic	21	27	.1	Motor aphasia	L lenticulostriatal
5	58	F	2	ischemic	47	23	0	None	L lenticulostriatal
6	45	F	4	hem	62	28	-1.18	None	R parietotemp
7	44	M	6	ischemic	54	29	-2.33	Conductive aphasia	L parietotemp
8	51	F	4	ischemic	6	17	-1.08	None	R parietotemp
9	64	M	5	hem	52	24	-0.2	None	R thalamus
10	61	M	7	ischemic	19	26	-1.28	Global aphasia	L frontotemp+capsula interna+putamen
11	59	M	3	ischemic	8	12	-2.37	None	R lenticulostriatal
12	62	M	2	ischemic	60	24	-2.37	None	R temporo-occip, thalamus

13	35	F	2	ischemic	20	16	-0.2	None	R lenticulostriatal, insular
14	62	M	1	ischemic	62	27		None	L putamen
15	41	M	7	ischemic	24	17	-0.5	None	L lenticulostriatal
16	67	M	3	ischemic	37	18		None	L cerebellum, medulla obl
17	53	F	2	ischemic	27	25	-1.65	Motor aphasia	L frontoparietotemp
18	53	M	3	hem	17	15	-1.41	None	R front, capsula interna
19	49	M	2	ischemic	62	29	-0.5	None	R frontopar, insular
20	63	F	4	ischemic	15	18	-1.08	None	R lenticulostriatal
21	17	F	3	hem	46	13	.39	Amnestic aphasia	L n.lentiformis, thalamus
22	35	M	9	hem	19	21	0	Verbal apraxia	L frontoparietal
23	62	M	2	hem	15	16	-0.1	N	L thalamus
24	37	M	8	hem	16	13	-.71	Motor aphasia, verbal apraxia	L frontoparietal
25	61	M	3	hem	17	23		None	R lenticulostriatal
26	53	M	2	ischemic			.1	None	L pons
27	68	M	2	ischemic	64	21	-1.48	None	R pons
28	48	M	7	hem	13	19	-2.05	None	R frontoparietotemp
29	60	M	12	hem	4	9	-1.18	None	R thalamocapsul
30	57	F	1	ischemic	54	25	-0.2	None	R frontal, insular
31	53	M	4	haem	42	21	-1.18	None	R parietal
32	60	M	1	ischemic	65	23	.31	None	L capsula interna
33	64	M	2	ischemic	15	12	.2	None	R pons
34	60	M	2	hem	10	17	-.2	Motor aphasia	L frontal
35	46	M	3	hem	17	14	.1	None	R lenticulostriatal
36	39	F	4	hem	4	7	-1.75	None	R frontoparietotemp
37	65	F	4	ischemic	39	17	-2.37	None	R frontopar, insular

TAP: test of attentional performance; M:male; F:female; L:left; R:right; hem: hemorrhagic

FM-UE: Fugl Meyer Scale- Upper Extremity; MF-LE: Fugl Meyer Scale-Lower Extremity
